# Immune Protection of Retroviral Vectors Upon Molecular Painting with the Complement Regulatory Protein CD59

**DOI:** 10.1007/s12033-016-9944-z

**Published:** 2016-05-11

**Authors:** Susanne Heider, Sandra Kleinberger, Feliks Kochan, John A. Dangerfield, Christoph Metzner

**Affiliations:** Institute of Virology, University of Veterinary Medicine, Veterinärplatz 1, 1210 Vienna, Austria; Anovasia Pte. Ltd., 3 Biopolis Drive, #05-19 Synapse, Singapore, 138623 Singapore

**Keywords:** Molecular Painting, Protein engineering, Gene therapy, Vaccination, Viral vectors, Complement, CD59

## Abstract

Glycosylphosphatidylinositol anchoring is a type of post-translational modification that allows proteins to be presented on the exterior side of the cell membrane. Purified glycosylphosphatidylinositol-anchored protein can spontaneously re-insert into lipid bilayer membranes in a process termed Molecular Painting. Here, we demonstrate the possibility of inserting purified, recombinant CD59 into virus particles produced from a murine retroviral producer cell line. CD59 is a regulator of the complement system that helps protect healthy cells from the lytic activity of the complement cascade. In this study, we could show that Molecular Painting confers protection from complement activity upon murine retroviral vector particles. Indeed, increased infectivity of CD59-modified virus particles was observed upon challenge with human serum, indicating that Molecular Painting is suitable for modulating the immune system in gene therapy or vaccination applications.

**Electronic supplementary material:**

The online version of this article (doi:10.1007/s12033-016-9944-z) contains supplementary material, which is available to authorized users.

## Introduction

Approximately 0.5 % of eukaryotic protein species carry a glycosylphosphatidylinositol (GPI) anchor [8]. Biochemically, the GPI anchor is characterized by an ethanolamine residue connecting the anchor to the C terminus of a protein, followed by a characteristic stretch of carbohydrate elements, mainly mannose residues [14] which in turn is connected to the phosphoinositol moiety with 2–3 lipid residues which are finally anchored in the outer leaflet of the plasma membrane [6]. Functionally, GPI-anchored proteins are diverse: they contribute to the regulation of the immune system (CD55, CD59), have enzymatic activities (alkaline phosphatase) or play roles in signal transduction (Thy-1) [13]. GPI anchoring is achieved by the transfer of preformed anchor structures onto proteins at the inner membranes of the endoplasmatic reticulum (ER) by the transamidase (TA) enzyme complex. Generation of the anchor requires the concerted activity of more than 20 enzymes [12]. GPI-anchored proteins may be released from cell membranes and shed into the surrounding media by different mechanisms: either as part of membrane-bound vesicles, such as exosomes, but also in forms excluding lipids both with absent or intact anchoring structures [1, 17, 20]. An interesting property of GPI-anchored proteins is that they are capable of re-inserting into lipid bilayer membranes [3–5, 7, 21, 22, 26, 29, 31–35]. This was described for the first time in 1984 for cell membranes under the term cell painting [21]. In 2008, we extended the system to include enveloped viral particles, originally of retroviral origin [29]. In 2013, also Influenza and herpesvirus particles were shown to be susceptible to Molecular Painting (MP) [26]. Infectivity of the virus is not necessarily inhibited by the modification itself [26, 29]; however, all post-exit incubation steps will reduce the viral half-life as a simple consequence of handling and exposure to ambient or elevated temperatures. Proteins may be converted into recombinant GPI-anchored proteins by introducing a C-terminal GPI signalling sequence (GSS) in addition to an N-terminal signal peptide (SP) [15, 16, 27, 28]. While the SP directs the nascent protein towards the ER, the GSS is recognized by the TA complex and transfer of the preformed GPI anchor can occur [28, 30]. In such a manner, for example, green fluorescent protein (GFP) [19, 26], CD4 [3] and interleukin-2 (IL-2) [15] have been converted into GPI-anchored proteins. Recently, artificially GPI-anchored proteins have been used for biomedical applications in vivo [32–34]. In contrast, CD59 (alternatively called protectin) is a naturally occurring GPI-anchored protein with functions in the regulation of complement activity. The complement system constitutes a part of the innate immune system that attacks pathogens upon triggering by various stimuli, mainly the generation of antigen–antibody complexes, but also a range of antibody-independent signals such as carbohydrate residues or different pathogen-derived proteins [11, 39, 41]. Enveloped viral particles are susceptible to inactivation by the complement [2, 11, 39–41], but may also subvert the system to promote acute or persistent infections [9, 11, 18]. Three different pathways of complement activity are described: the classical, alternative and lectin pathways [23]. These pathways show a considerable overlap and lead to the same effector mechanism. The initiator protein of the classical pathway, C1q, interacts with more than 100 different proteins, including a range of viral envelope glycoproteins [41]. Additionally, the main initiator protein of the lectin pathway, mannose-binding lectin MBL, also binds to viral surface markers, e.g. of human immunodeficiency virus type 1 (HIV-1) [41]. From a biochemical viewpoint, proteolytic cascades are initiated (for a recent, comprehensive review see [23, 24]) leading to membrane deposition of enzymatically active protein complexes (“convertases”) and the formation of soluble pro-inflammatory intermediates, termed anaphylatoxins. Effector mechanisms of the complement system include the generation of said anaphylatoxins and the formation of lytic pores in lipid membranes through formation of the membrane attack complex (MAC). Additionally, opsonization of pathogens allows for a more efficient recognition, uptake and processing of antigens by professional antigen-presenting cells. A tight regulation of the complement system is necessary to avoid prolonged or unwarranted activation. A whole range of regulatory molecules (both soluble and membrane-bound), including CD46, CD55 and CD59, are involved in controlling the complement system. CD59 inhibits formation of the MAC by preventing assembly of sufficient C8 and C9 late complement proteins [10]. As a means of immune evasion, lentiviral particles include complement regulatory proteins into their envelope when budding from the cell, thus protecting them from the complement system [36, 37]. Artificial display of CD59 on viral particles after transfection of murine virus-producing cell lines also induced protection of viral particles from the complement [2, 38]. In this study, we proposed to protect retroviral vector particles produced from a non-human source, i.e. mouse-derived PALSG/S cells [2], from the human complement system by means of MP (rather than transfection) to provide proof-of-principle that functional surface engineering of vectors via MP may be employed for fine-tuning of viral vector properties, e.g. for future use in human gene therapy or vaccination approaches.

## Material und Methods

### Cells/Plasmids/Virus/Serum

CrFK, HeLa and NIH3T3 cells were acquired from ATCC stocks (Accession numbers CCL94, CCL-2 and CRL-1658, respectively). The generation of the expression plasmids coding for CD59his (pCD59hisneo) and the respective expressing cell lines has been described previously [29]. The murine leukaemia virus (MLV)-based vector particles pseudotyped with 4070A amphotropic Env were produced from the stable murine producer cell line PALSG/S [2] based on the murine fibroblast cell line NIH3T3. All cells were cultured in DMEM supplemented with 10 % foetal calf serum (FCS, purchased from BioChroma). Cells transfected with pCD59hisneo were kept under selection in 400 µg/ml G418 (Sigma-Aldrich). Human serum was either purchased in lyophilized form (Sigma-Aldrich) or prepared from healthy human donors. Preparation of serum and separation from cellular proteins were carried out using BD Vacutainer^R^ Rapid Serum Tubes (Becton–Dickinson). In brief, after adding the whole blood to the tubes, blood rested for 10 min at room temperature and was centrifuged for 10 min at 1500×*g*. The serum was removed from the top of the tubes and stored in aliquots at −80 °C until use. In both cases, heat inactivation was carried out at 56 °C for 30 min.

### Virus Harvesting

PALSG/S cells were cultured in DMEM (Life Technologies) containing 10 % FCS (BioChroma). 72 h prior to harvesting of the viral supernatants, cells were transferred into serum-free DMEM. Supernatants were centrifuged for 10 min at 2300×*g* before filtration through a 0.45-µm syringe filter. Finally, supernatants were subjected to ultracentrifugation in a Beckman ultracentrifuge using an SW32TI rotor at 54,000×*g* (at average radius) for 2 h at 4 °C. The supernatants were discarded, and the pellets were resuspended in DMEM and stored at −80 °C until further use.

### Protein Purification

A fast protein liquid chromatography (FPLC) device (ÄktaPrime Plus, GE HealthCare) using ready-made immobilized metal affinity chromatography (IMAC) columns (HisTrap FF crude, GE Healthcare) was employed for protein purification. Cells were washed in PBS and scraped into PBS. After collecting cells by centrifugation, sample application buffer (SAB, containing 50 mM Tris–HCl, 50 mM NaCl, 35 mM imidazole and 1 % (w/v) octylglucoside (OG), pH 7.4) was added. During the FPLC procedure, wash buffer (WB, containing 50 mM Tris–HCl, 50 mM NaCl, 35 mM imidazole, pH 7.4) was used and attached proteins were eluted through a continuous gradient ending in a final concentration of 100 % elution buffer (EB, containing 50 mM Tris–HCl, 50 mM NaCl, 600 mM Imidazole, pH 7.4). Following purification, elution fractions were analysed by UV spectrometry and immunoblotting. Positive fractions were collected for further concentration using ultrafiltration columns (Vivaspin 20 Turbo, Sartorius Stedim, molecular weight cut-off 10 kD) and washed twice in 10 ml protein storage buffer (50 mM Tris–HCl, 50 mM NaCl, pH 7.4), then stored at −20 °C until further use. Total protein content of the preparations was determined by a modified Lowry assay (BioRad Protein DC kit, according to manufacturer’s instructions). The presence of proteins of interest was confirmed by immunoblotting.

### Immunoblots

Samples were subjected to SDS-PAGE using 10 % gels and a Laemmli buffer system. Proteins were electro-transferred onto PVDF membranes (GE Healthcare) and incubated overnight in blocking buffer (4 % milk powder w/v; 1 % bovine serum albumin in Tris-buffered saline containing 0.1 % Tween-20). Primary antibodies were used in 1:2000 dilutions (for CD59). Antibodies directed against CD59 were purchased from AbD Serotec. Rat anti-MLV capsid and envelope antibodies were purified by Biomedica (Vienna). HRP-labelled anti-murine and anti-rat secondary antibodies (DakoCytomation) were used in dilutions of 1:5000 and 1:10,000, respectively. ECL detection kits (GE Healthcare) were used for generating signals, which were developed and recorded using an AGFA Curix 60 developer and Hyperfilm ECL (GE Healthcare).

### Cellular Serum Response

HeLa, CrFK, CrFK-CD59his and PALSG/S cells were seeded in triplicate at the densities of 10^4^ cells per well in 96-well plates. 24 h later, cells were treated with DMEM containing 50 % active human serum or 50 % inactivated human serum. After 24 h of incubation, the cells were trypsinized and 50 µl of the cell suspension was used for manually counting in a counting chamber (MEGUMED Diagnostik GmbH, Germany).

### Molecular Painting

MP reactions were set up in a total volume of 500 µl. The final concentration of CD59his in MP reactions was 35 ng/µl. After incubation of samples for 120 min at 37 °C under constant shaking, non-associated protein was removed by ultracentrifugation as described for the virus harvesting earlier. Samples were resuspended in 200 µl DMEM.

### Serum Treatment and Infections

For infection, HeLa cells were seeded in 6-well plates at a density of 1.5×10^6^ cells per well 24 h prior to infections. After MP, 200 µl of painted virus was divided in half and mixed with either 400 µl active human serum or heat-inactivated human serum. After 1-h incubation at 37 °C, 5 % CO_2_ under constant agitation samples were added dropwise onto seeded HeLa cells with additional hexadimethrine bromide (Sigma-Aldrich) yielding a final concentration of 8 µg/ml to overcome charge repulsion of virus and cell membranes. After 30-min incubation, 2 ml of DMEM +10 % FBS and additionally 20 µl hexadimethrine bromide were added dropwise onto HeLa cells (see Sect. 2.9). The cells were incubated at 37 °C, 5 % CO_2_ until flow cytometry analysis was carried out.

### Flow Cytometry

72 h post infection, cells were washed in PBS once before being removed from the plates by trypsin. Samples were collected by centrifugation for 5 min at 200×*g*, washed in PBS and centrifuged as described previously. Finally, the cells were fixed using 4 % formalin in PBS, filtered and analysed on a BD cytometer using CellQuest software.

### Calculations and Statistical Analysis

Cellular survival rates (CSR, Fig. 3) were calculated as the number of cells in the samples treated with active serum multiplied with 100, then divided by the number of cells in the samples treated with inactivated serum. The virus protection factor (VPF, Fig. 4) indicates to what extent a treatment (i.e. MP with CD59his) allows the virus to better withstand serum complement activity. VPF is calculated as the ratio of the relative virus survival compared to a reference (i.e. non-treated). The relative virus survival is calculated in a similar manner to the CSR: as the percentage of infected cells (showing green fluorescence) incubated with virus samples treated with active serum multiplied with 100, then divided by the percentage of infected cells incubated with samples treated with inactivated serum. Student’s *t* test (two-sided, paired) was used in all cases to determine whether averages are significantly different between treated and untreated groups.

## Results

### Expression and Purification

**Fig. 1:**
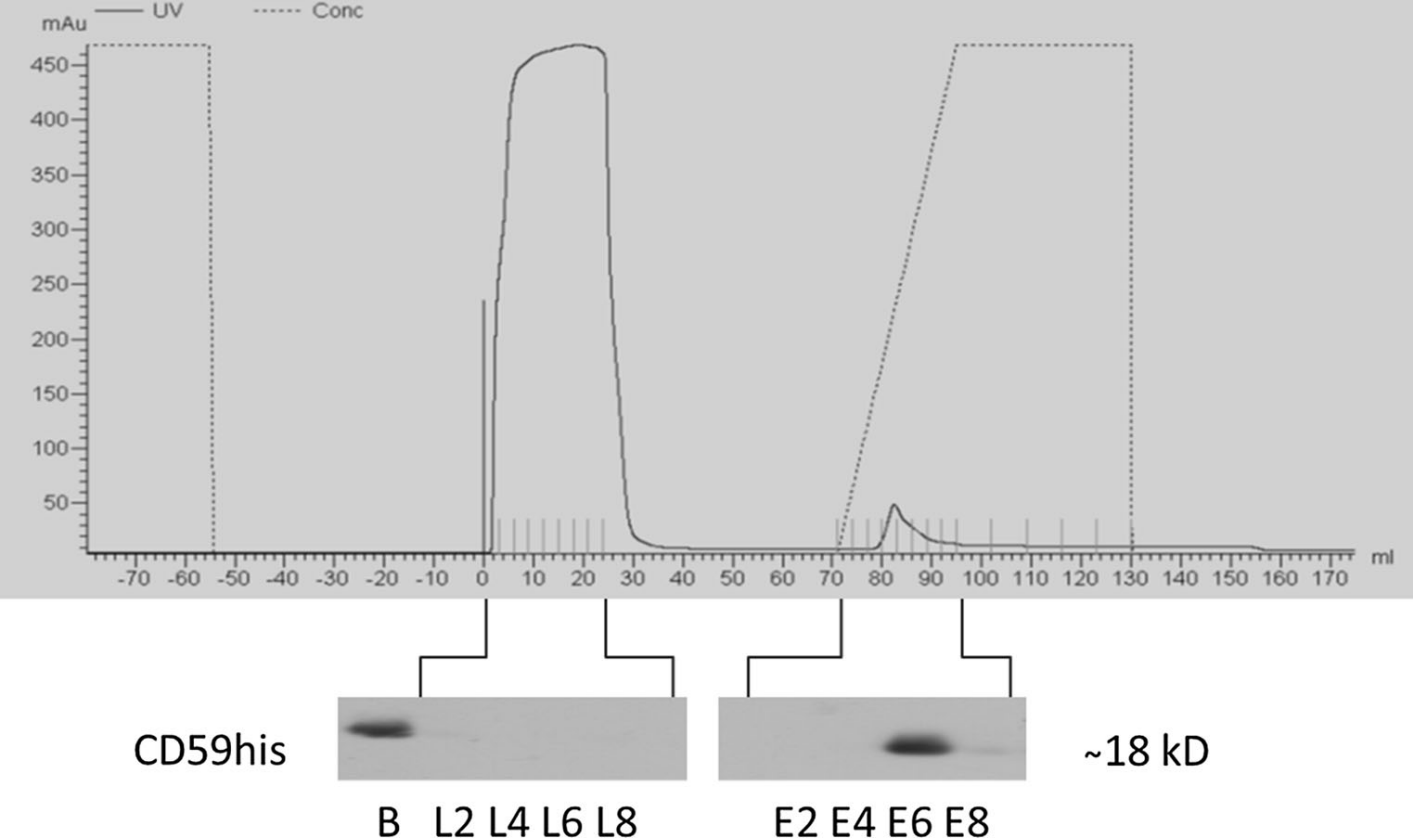
Purification of GPI-anchored proteins. The *figure* shows a representative result from a series of purification runs. Total cell extracts were purified using immobilized metal affinity chromatography. The *upper section of the figure* shows the total protein content of the mobile phase at any given time during the protocol (*black line*) and the relative concentration of eluent (*dotted line*). The *vertical lines* separate the fractions taken. In the *lower part*, fractions taken from sample loading (*L2*–*L8*) and elution (*E2*–*E8*) were analysed by immunoblotting using antibodies directed against CD59 to specifically detect the purified proteins. Samples taken before application to the column (*B*) serve as protein expression control

The sequence of human CD59 was modified by genetic engineering to contain a run of 6 histidine residues (the his-tag), respectively, and transfected into the feline cell line CrFK [29]. Expression was confirmed after selection by immunoblots using specific antibodies (Fig. 1, bottom, samples labelled “B”). Purification of CD59his was subsequently performed using an FPLC approach as previously described [26, 29]. Fractions were collected before purification (Fig. 1, bottom, samples labelled “B”), during sample loading onto the column and elution. Samples corresponding to the eluted protein peaks from the FPLC UV trace (Fig. 1, top, black line) were collected and subjected to ultrafiltration for buffer exchange and concentration. Signals are found in the starting material (Fig. 1, bottom, samples B) and elution fractions (Fig. 1, bottom, samples E2–E8) upon increase in the concentration of imidazole (Fig. 1, top, black dotted line), as expected.

### Molecular Painting

**Fig. 2:**
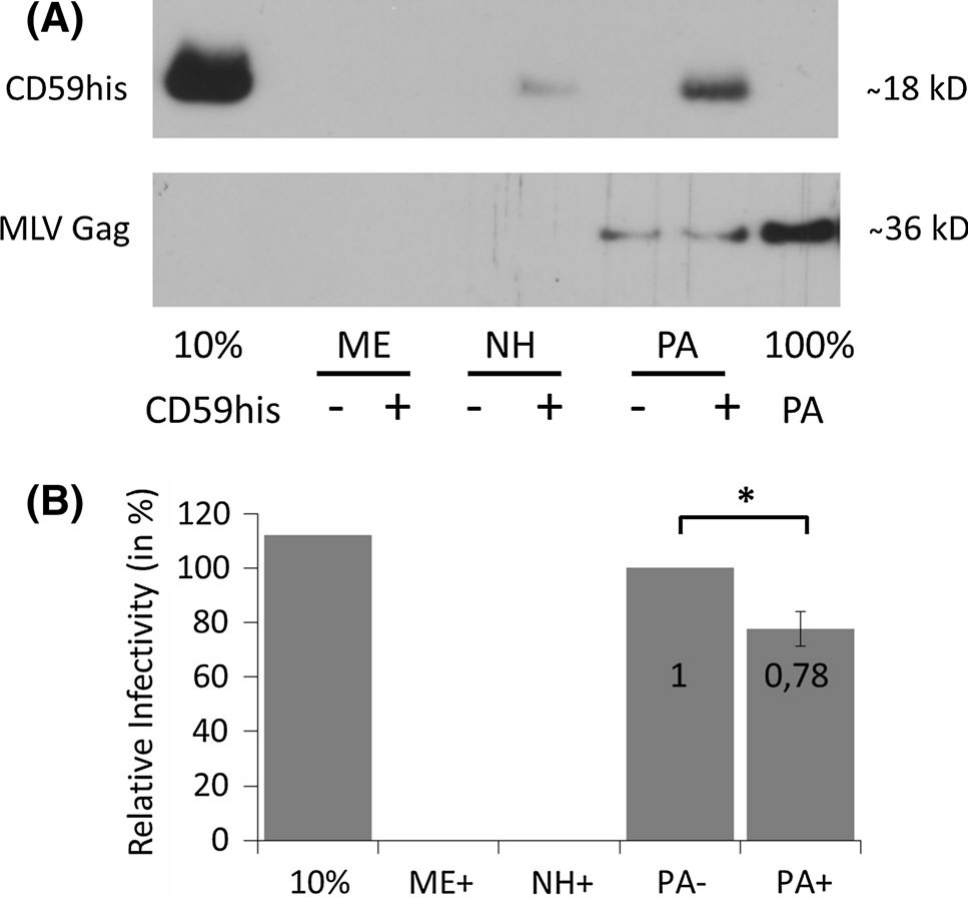
Overview of Molecular Painting. The *graph* shows an overview of the MP procedure necessary for carrying out the complement protection studies. **a** MP with CD59his. Signals are found if GPI-anchored protein and viral particles are present in the reaction mix (samples labelled PA+), but neither in the absence of virus (samples labelled ME+) nor in the absence of GPI-anchored proteins (samples labelled PA−). When concentrated supernatant from the non-virus-producing parental cell line NIH3T3 was used for MP, a faint band is visible in the presence of CD59his (sample NH+). Control samples indicate the signals corresponding to 10 % CD59his and 100 % virus input (10 % CD59his and 100 % PA, respectively). B depicts relative infectivity (transduction rates) from MP-treated samples. Samples containing only medium or concentrated supernatant from NIH3T3 do not contribute to infectivity (samples ME+ and NH+; for clarity samples ME− and ME− are omitted). Infection was observed for both viral particle-containing samples PA− and PA+. A small, but significant drop in infectivity was observed from PA− to PA+ (factors are *inset* into *columns*). As a transduction control, ten percent of untreated virus input was used for infection (sample 10 %). Results represent average and standard deviation from two independent experiments. *Asterisks* indicate statistically significant differences between groups

Subsequently, we were interested in the recombinant proteins’ ability to perform in MP. For CD59 and MonoGGhis, MP had been reported previously onto lentiviral and retroviral particles [26, 29], however not specifically onto particles produced from the producer cell line PALSG/S (derived from murine NIH3T3 cells). CD59his could also be successfully attached to viral particles produced from these cells (Fig. 2a). This further indicates that the provenance of the viral envelope lipid bilayer does not influence MP significantly. No signal could be detected when either no virus, or no CD59his was provided (Fig. 2, panel A, bottom, samples ME+ and PA−, respectively). Only a very weak attachment could be observed when the parental cell line NIH3T3 was used (Fig. 2, panel A, top, sample NH+). The presence and the amount of virus were controlled by immunoblotting specific for the MLV Gag proteins. All concentrated cell culture supernatants were collected from comparable amounts of producing cells.

In previous publications, no significant decrease in infectivity induced by MP was observed, but a rather strong loss of virus (activity) as a result of the duration of the process [29]. We could confirm this time-dependent decrease (Fig. 2a, compare signals in MLV Gag panel in lanes PA−, PA+ and 100 % PA and Fig. 2b, compare samples 10 %, PA− and PA+). However, additionally we observed a small but statistically significant drop in infectivity between mock-painted and CD59-painted samples (Fig. 2b, compare samples PA− and PA+).

### Cellular Response

**Fig. 3:**
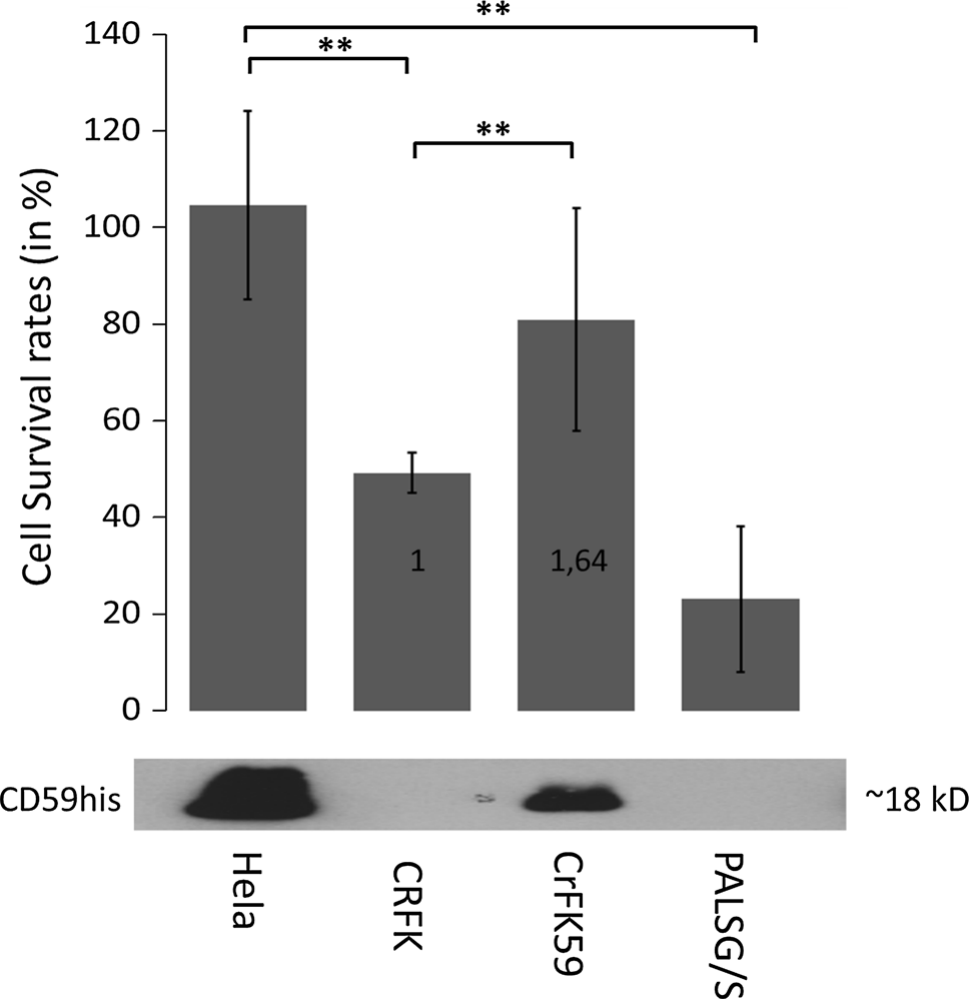
Cellular responses to serum treatment. The *bottom section* shows the expression of complement regulatory proteins in the cell lines HeLa, CrFK, CrFK stably transfected with CD59his and the virus-producing cell line PALSG/S. The *upper section* shows the survival rates of the cell lines after incubation with active human serum compared to inactivated serum. The ratio of protection between CrFK cells not expressing/expressing is *inset* in the *columns*. *Columns* and error bars represent means and standard deviations, respectively. Means and standard deviations were calculated from at least four independent experiments. Additional statistical information is summarized in Supplementary Table 1. *Asterisks* indicate statistically significant differences between groups (*p < 0.05; **p < 0.01)

Before analysing the response of viral particles treated by MP with complement regulatory protein to complement, we were looking at cell lines transfected with said complement regulatory proteins, mainly to see if the recombinant proteins would protect from complement in a heterologous background. Additionally, we were interested to see whether the cell membranes, giving rise to the viral envelopes, are sufficiently susceptible to complement attack in this setting. HeLa, CrFK, PALSG/S and CrFK cells stably expressing CD59his were subjected to treatment with active and heat-inactivated serum, and their numbers followed over a period of 24 h post exposure (Fig. 3). Expression of the complement regulatory factor CD59 is demonstrated by immunoblotting from cell extracts of equal total protein amounts (Fig. 3, bottom panel). HeLa cells endogenously express CD59 and thus could serve as a positive control for complement protection. The human cell line HeLa indeed showed an increased growth (CSR > 100 %) when cultured with active human serum, most probably due to the availability of fully active species-specific growth factors in serum. CrFK and PALSG/S cells were not expressing CD59. Both cell lines were more susceptible to complement lysis, with the strongest effect being seen in the cell line used for virus production (PALSG/S). CrFK cells expressing CD59his after stable transfection showed an increased resistance to complement activity (Fig. 3, compare samples CrFK and CrFK59). When performing statistical analysis of the results, cell survival rates (CSR) for CrFK cells CD59his were not statistically significantly different from those observed for perfectly protected HeLa cells, but quite decidedly different from effects on PALSG/S cells which provide the membranes for viral envelopes (see supplementary table 1).

### Virus Response

**Fig. 4:**
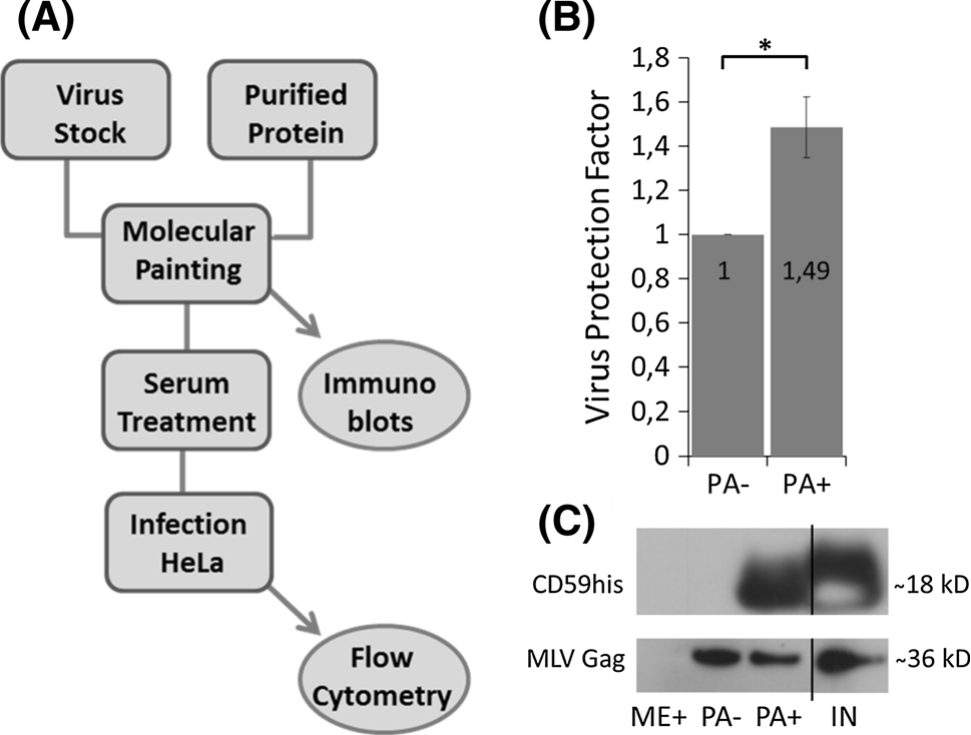
Viral responses to serum treatment. **a** The scheme shows an overview of the experimental procedures leading to the results presented in the graph in **b**. **b** Virus particles derived from PALSG/S cells subjected to MP with CD59 were incubated with active or inactivated human serum and finally used to infect HeLa cells. 72 h post infection, cells were harvested and analysed by flow cytometry. *Columns* and *error bars* represent means and standard deviations, respectively. Shown are protection factors, showing the relative increase of virus survival compared to mock-treated virus particles (numerical values *inset* in *columns*). *Asterisks* indicate statistically significant differences between groups. *Columns* and *error bars* represent means and standard deviations, respectively. Means and standard deviations were calculated from three independent experiments. Viral particles were protected better from serum complement activity after MP with CD59his. **c** Immunoblots were carried out to control for successful painting (CD59his signal only visible in the presence of virus and GPI-anchored protein not in samples containing no virus or no protein; compare samples PA+, ME+ and PA−) and virus amounts (comparable levels of MLV Gag protein were found in PA− and PA+ samples). IN signifies the 10 % untreated input level

Finally, we were interested to see whether MP of viral particles could provide protection from the complement activity of human serum. Virus particles painted with CD59his or mock-painted were subjected to treatment with either active or heat-inactivated human serum. Virus samples were then used for infection of HeLa cells and analysed by flow cytometry 72 h post infection (for an overview of the experimental procedure see Fig. 4a). The ratio of viruses that remained infectious after treatment with active human serum (compared to treatment with inactivated serum) was increased for virus painted with CD59his (Fig. 4b). A VPF of 1.49 was observed, corresponding to 49 % more virus particles available for infection, for viruses treated with CD59his. The VPF indicates to what extent a treatment (i.e. MP with CD59his) allows the virus to better withstand serum complement activity. When a non-complement regulatory protein—the monomeric GFP variant MonoGGhis—was used for MP, no such effects were seen (see supplementary figure 1). VPF is calculated as the ratio of the relative virus survival compared to a reference (i.e. non-treated). The relative virus survival is calculated as the percentage of infected cells (showing green fluorescence) incubated with virus samples treated with active serum multiplied with 100, then divided by the percentage of infected cells incubated with samples treated with inactivated serum.

## Discussion

In this study, a different virus-producing cell line was subjected to MP than previously reported: the murine NIH3T3-based PALSG/S cell line. When comparing MP results from these to previously reported MP of virus derived from human cell lines [29], an interesting effect was observed. Supernatants derived from the non-virus-producing cell line NIH3T3 were also amenable to MP (see Fig. 2, samples NH− and NH+). This is most likely a result of concentration of membrane vesicles such as exosomes during preparation. As a consequence, MP may also be developed for applications on exosomal preparations. Also, no significant difference in infectivity was observed between MP- and mock-treated virus preparations in previous experiments [29]. In this study, we could identify a small, but statistically significant decrease in infectivity with CD59his-treated virus as compared to mock-treated one (see Fig. 2b, samples PA+ and PA−). The different virus-producing cell lines used (HEK293T based in earlier experiments, NIH3T3 based in this experiments) may thus have different sensitivities. Alternatively, this decrease may depend on the efficiency of protein insertion, i.e. a certain density of inserted GPI-anchored proteins may interfere with virus binding and/or fusion. Interestingly, expression of CD59his failed in PALSG/S and the parental NIH3T3 cells, even after repeated attempts using different transfection protocols. This is unfortunate because viruses harvested from these transfected producer cell lines would have provided an interesting control for determining efficacy of MP when compared to transfection-based approaches. Also, the maximum protection that would have been achieved in these cells by transfection with CD59his would have been interesting. In general, the reaction of cell lines to human serum was mostly as expected. HeLa cells, which are of human origin (expressing CD59), were fully resistant to human complement. CrFK and PALSG/S (lacking expression of CD59) demonstrated varying degrees of complement lysis, with the stronger effects observed in PALSG/S (Fig. 3, top). This may be due to the fact that while both cell lines are of non-human origin (feline and murine, respectively) and will thus carry complement-stimulating determinants, PALSG/S cells also express viral antigens, which may lead to a more efficient triggering of the complement system [39, 41]. CrFK cells expressing CD59his showed an intermediate response (i.e. partial protection, protection factor of 1.64 compared to non-expressing) to active serum (see Fig. 3). This may indicate the need for cooperation between two or more regulatory factors to confer full protection. Alternatively, expression may be too low or protein functions reduced as a result of introduction of the his-tags. Taken together, these results suggested that virus produced from PALSG/S cells is susceptible to complement activity, even in the absence of specific antibodies, and furthermore that recombinant CD59his is able to provide at least partial protection from the complement system. Both conclusions are important prerequisites for determining effects of MP with complement regulatory factors on viral vectors. Upon modification by MP, virus samples were subjected to active human serum and then used to infect HeLa cells (see Fig. 4). We were interested to see if modified virus would be more competent in withstanding complement attacks, indicated by the ratio of infected cells after treatment with active vs. inactive serum, i.e. the VPF (see Fig. 4). Indeed, viral particles modified with CD59his were better able to withstand complement activity than mock-treated virus particles or particles modified with the control GFP-based GPI-anchored protein, MonoGGhis (see supplementary figure 1). The VPF, i.e. the multiple of protection introduced by CD59 modification in comparison to mock-treated viral particles, achieved by MP seems low, with the CD59his modification giving a virus protection factor of 1.49 (see Fig. 4). However, in practical terms, this means that 49 % more virus is available for productive infection. This may very well prove relevant for use in gene therapy applications. Several reasons will contribute to the protection being only partial. Introduction of GPI-anchored protein may negatively affect infectivity by default (see Fig. 2, samples PA− and PA+). When correcting for this effect, nearly 100 % more virus is available for infection. Also introducing MonoGGhis by MP is reducing serum protection. Reduction levels were strikingly similar (0.78 for general infectivity reduction by MP in the absence of active serum and 0.82 for MP with MonoGGhis in the presence of active serum). This may indicate that the reduction seen upon MP with MonoGGhis is more likely an effect of loss of infectivity by GPI incorporation rather than increased stimulation of the complement system by MonoGGhis. In the physiological situation in vivo, more than one regulatory protein is interfering with complement proteins to limit their activity. We tried to mimic this situation by double MP with CD55 (also termed decay accelerating factor, another GPI-anchored regulator of complement activity) and CD59. However, MP with CD55 yielded very little modification, most likely as a result of competition for available membrane space on the viral envelope. When comparing the protection achieved in cells and virus preparation, the protection factors are similar, i.e. 1.64 for cells (see Fig. 3) and 1.49 (corrected 1.91) for virus (see Fig. 4). This suggests that the partial protection is rather a result of the protein function than the MP process, i.e. defective or incomplete GPI processing may be responsible for the small protection factor, as well as mis-folding at the protein level of the CD59his molecule. On a more general level, complement activity in the circumstances used in this study will be mostly triggered by the classical pathway in an immune complex independent manner [23, 24] since all complement assays have been performed in the absence of antibodies. Also the lectin pathway may be triggered by the presentation of unusual carbohydrates on the murine- and feline-derived envelope structures [23, 24]. This suggests that viral vectors produced in human cells may also be susceptible to inactivation by the complement system, if insufficient amounts of complement regulatory proteins are present without modification by MP.

GPI-anchored proteins are notoriously difficult to work with: usually expression levels are low, even when using strong promoters for driving expression. The low abundance mostly arises from the limitation of the GPI anchor biosynthesis pathway of cells in general. Additionally, the strong inherent amphiphilic nature of the GPI anchoring may promote aggregation events or the formation of pseudo-micelles, which could in turn contribute to poor performance in affinity purification. As a result, purity is poor (approximately 1 % as measured by his-tag ELISA, data not shown).

In this study, we could demonstrate that modification of retroviral vector particles with a regulator of the complement system using MP leaves them less susceptible to complement attack. On a more general note, this provides further evidence for the usefulness of MP in a range of biomedical applications, from labelling of virus particles for research purposes [26] and vaccination strategies using VLPs [32, 34] to fine-tuning vector properties for gene therapy applications [25, 27, 28, 30].

## Electronic supplementary material

Below is the link to the electronic supplementary material.

Supplementary material 1 (DOCX 188 kb)

## Abbreviations

CSR: Cellular survival rates
GPI: Glycosylphosphatidylinositol
GSS: GPI signalling sequence
MP: Molecular Painting
VPF: Virus protection factors
