# Supplementary material for: Immune Protection of Retroviral Vectors Upon Molecular Painting with the Complement Regulatory Protein CD59
Source: Mol Biotechnol. 2016 May 11;58:480–8. doi: 10.1007/s12033-016-9944-z (PMC4899500; doi:10.1007/s12033-016-9944-z)
Supplement: Supplementary file 1 — Supplementary material 1 (DOCX 188 kb) [file 12033_2016_9944_MOESM1_ESM.docx]

Supplementary Material and Methods. The preparation of the monomeric GFP variant monoGGhis was reported previously ([Metzner et al., 2013a](#_ENREF_26)). MP was performed using the same amount of virus particles as for CD59his-painting and the same concentration of GPI anchored protein (35 ng/µl total protein). Incubation, subsequent purification treatments and serum treatment were the same as described for CD59his MP.

|  | PALSG/S | CRFK | CRFK59 | HELA |
| --- | --- | --- | --- | --- |
| PALSG/S | x | **0,0037** | **0,0015** | **4,87E-05** |
| CRFK |  | x | **0,0088** | **3,42E-05** |
| CRFK59 |  |  | x | 0,1151 |
| HELA |  |  |  | x |

Supplementary Table 1. **Statistical analysis of cellular responses to serum treatment – Summary of p–values.**

**
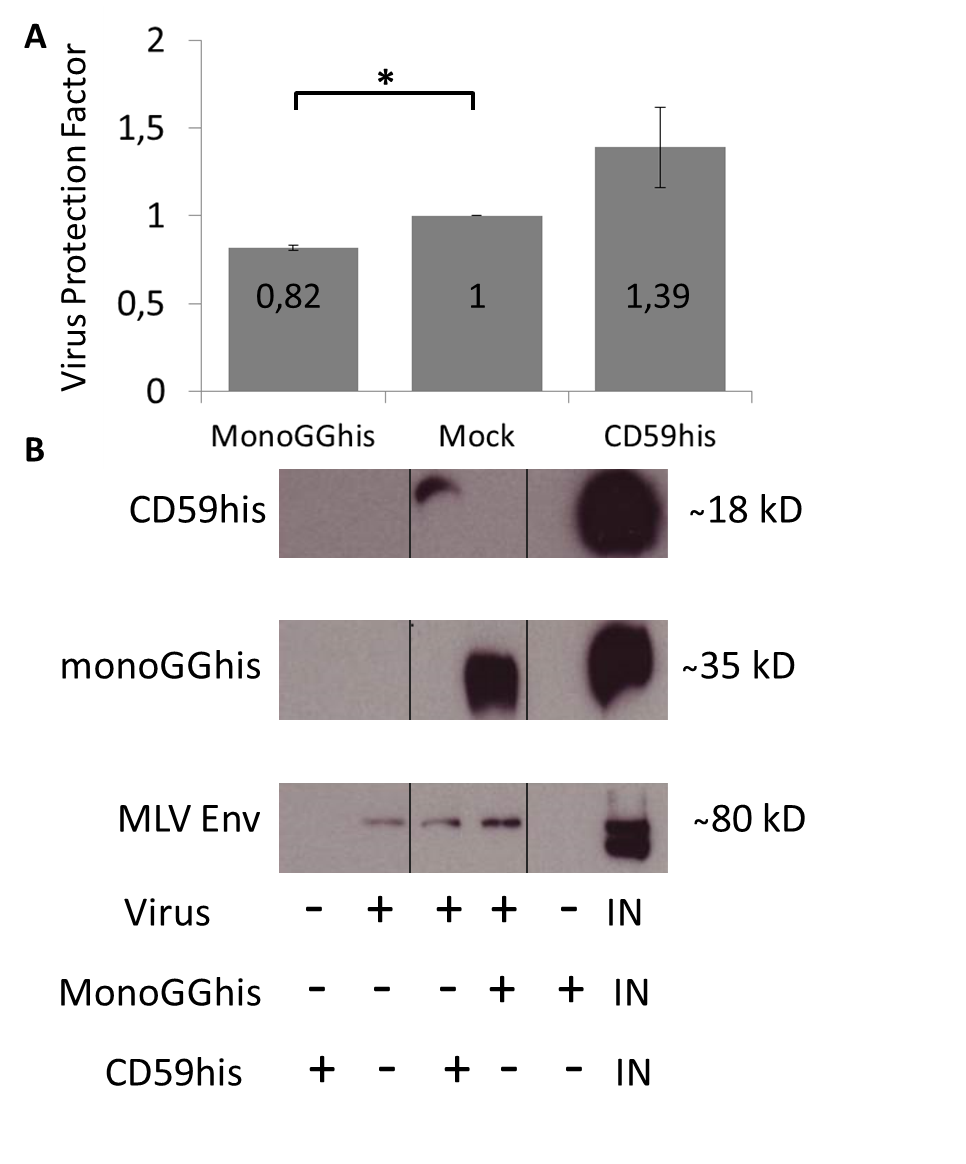
**

Supplementary Figure 1. **Molecular painting with GPI anchored monomeric GFP.**

A Virus particles derived from PALSG/S cells subjected to MP with CD59 or monoGGhis were incubated with active serum and finally used to infect HeLa cells. 72 hours post infection, cells were harvested and analysed by flow cytometry. Columns and error bars represent means and standard deviations, respectively. Shown are protection factors, showing the relative increase of virus survival compared to mock-treated virus particles (numerical values inset in columns). Asterisks indicate statistically significant differences between groups. Columns and error bars represent means and standard deviations, respectively. Means and standard deviation were calculated from 2 independent experiments. Viral particles were protected better from serum complement activity after MP with CD59his, confirming earlier results. MP with monoGGhis reduced serum protection when compared to mock treated samples. C Immunoblots were carried out to control for successful painting (CD59his signal only visible in the presence of virus and GPI anchored protein not in samples containing no virus or no protein) and virus amounts (comparable levels of MLV Gag protein were found in CD59his and MonoGGhis - and + samples). IN signifies the 10% untreated input level.
